# Melittin-Based Nano-Delivery Systems for Cancer Therapy

**DOI:** 10.3390/biom12010118

**Published:** 2022-01-12

**Authors:** Anqi Wang, Yuan Zheng, Wanxin Zhu, Liuxin Yang, Yang Yang, Jinliang Peng

**Affiliations:** School of Pharmacy, Shanghai Jiao Tong University, Shanghai 200240, China; wanganqi_5227@sjtu.edu.cn (A.W.); zhengyuanz@163.com (Y.Z.); isamuel@sjtu.edu.cn (W.Z.); yanglx_12@126.com (L.Y.); yangyang023@sjtu.edu.cn (Y.Y.)

**Keywords:** melittin, hemolysis, stable loading, nano-delivery system, tumor therapy

## Abstract

Melittin (MEL) is a 26-amino acid polypeptide with a variety of pharmacological and toxicological effects, which include strong surface activity on cell lipid membranes, hemolytic activity, and potential anti-tumor properties. However, the clinical application of melittin is restricted due to its severe hemolytic activity. Different nanocarrier systems have been developed to achieve stable loading, side effects shielding, and tumor-targeted delivery, such as liposomes, cationic polymers, lipodisks, etc. In addition, MEL can be modified on nano drugs as a non-selective cytolytic peptide to enhance cellular uptake and endosomal/lysosomal escape. In this review, we discuss recent advances in MEL’s nano-delivery systems and MEL-modified nano drug carriers for cancer therapy.

## 1. Introduction

MEL is a major component of honeybee (*Apis mellifera*) venom with various biological and pharmacological properties [1]. Its strong surface activity on lipid membranes, anti-microbial, anti-inflammatory, and anti-cancer properties has been widely studied and proved. However, the use and application of MEL in clinical experiments is hindered because of its intense toxic side effects. Multiple strategies have been adopted and optimized to develop a safe and stable MEL delivery system. This review focusses on the recent progress of MEL carriers and drug delivery systems with MEL as a functional molecule in cancer therapy. Measures to lower the side effects of MEL are also discussed, along with the improvements and challenges relevant to each strategy.

### 1.1. Structure of Melittin (MEL) and Its Interactions with Membrane

**Figure 1:**
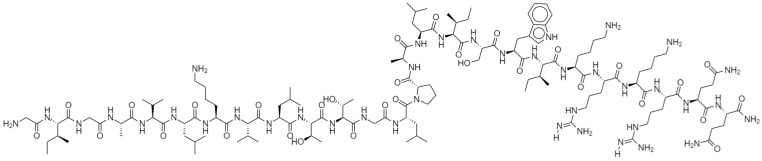
Chemical structure of MEL.

MEL was originally isolated and purified from bee venom (BV), and is the main active ingredient in BV, accounting for approximately 50% of its dry weight [1]. Briefly, MEL has been fractionated and isolated from BV through gel filtration, high-performance liquid chromatography (HPLC), or capillary electrophoresis (CE), and analyzed by ultraviolet assay, reverse-phase HPLC and amino acid analysis [2,3]. This 26-amino peptide (Figure 1) is sequenced as follows: Gly Ile-Gly Ala-Val-Leu-Lys-Val-Leu-Thr-Thr-Gly Leu-Pro-Ala-Leu-Ile-Ser-Trp-Ile-Lys-Arg-Lys-Arg-Gln-GlnNH2 [4]. Its N-terminal region is hydrophobic, and its C-terminal region is hydrophilic because of the presence of positively charged amino acids [5], which carry a net charge of +6 at physiological pH, leading to its amphiphilic property. Under natural conditions, MEL is in a tetrameric state and dissociates into monomers during ion intensity change [6,7]. The α-helix of MEL is an essential structure that produces its lytic effects. Under different physiological conditions, the α-helix forms a perpendicular or parallel conformation to the membrane surface. When anchored parallel to the membrane surface, the MEL molecule is inactive, and this inserting form prevents other peptides from inserting into the lipid bilayer. In the other case, MEL is inserted perpendicularly into the lipid bilayer and causes pore formation and membrane rupture, leading to the leakage of hemoglobin or other intracellular contents [8,9]. However, the specific mechanism of MEL and cell membrane remains controversial, as one study pointed out that the amphiphilic α-helix structure is formed after MEL insertion to cell membrane [10].

### 1.2. Pharmacological Effect of MEL

In addition to its strong surface activity on cell lipid membranes and hemolytic activity [11], MEL has tremendous biological and pharmacological effects, including anti-bacterial [12], anti-virus [13], anti-fungal [14,15] (Table 1), anti-inflammatory [16], and anti-tumor properties [4] (Table 2).

The anti-microbial effects of bee venom (BV) and MEL have been reported since the 1940s [27]. With growing concerns regarding drug-resistant bacteria, MEL has become a promising efficacious agent due to its properties of pore formation and bacterial destruction [28]. Evidence has confirmed the anti-bacterial effect of MEL, especially against drug-resistant bacteria that conventional antibiotics fail to inhibit or kill [20,24]. The combination of MEL with other antibiotics is widely evaluated [23,29]. One study evaluated MEL against methicillin-resistant *Staphylococcus aureus* (MRSA) strains [21] and reported a minimum inhibitory concentration (MIC) and minimum bactericidal concentration (MBC) of 6.7 and 26 μg/mL, respectively. Meanwhile, the MIC and MBC values for oxacillin were both 32 μg/mL. MEL in combination with oxacillin also showed synergistic results on MRSA strains.

MEL can also inhibit various viruses [13,30], including human immunodeficiency virus (HIV) [17,31], herpes simplex virus (HSV) [32,33], and respiratory syncytial virus (RSV) [13]. Its mechanism may include suppressing the gene expression of virus and impeding the multiplication process [17,18]. As an antiviral peptide, MEL is also a potential candidate against severe acute respiratory syndrome coronavirus 2 (SARS-CoV-2) [34,35]; a survey in beekeepers revealed the potential preventive effect of BV on coronavirus disease 2019 (COVID-19) [36]. A Sitagliptin (SIT)-MEL nano-conjugate was developed [19], and showed better antiviral potential against SARS-CoV-2 isolates than SIT and MEL alone, thus proving MEL to be one of the promising candidates.

In traditional medicine, BV has been used in the treatment of inflammatory-related illnesses [37]. MEL binds non-competitively with phospholipase A2 (PLA2) to inhibit its enzymatic activity, and thus can be used for the treatment of inflammation caused by the production or enhanced activity of secreted PLA2 [38]. MEL also has inhibitory effects on sodium nitroprusside, IκB kinase (IKK) activity, nitric oxide (NO), inducible NO synthase (iNOS), cyclooxygenase-2 (COX-2), and other inflammatory mediators due to its high binding affinity with IKKs [39], which suppresses IκB degradation and thereby blocks the nuclear factor kappa-light-chain-enhancer of the activated B cells (NF-κB) signaling pathway. MEL can inhibit the signaling pathways of toll-like receptor 2 (TLR2), TLR4, CD14, NF-κB essential modulator (NEMO), and platelet-derived growth factor receptor beta (PDGFRβ) [40,41,42,43], consequently reducing the activation of p38 mitogen-activated protein kinases, extracellular signal-regulated protein kinase (ERK1/2), protein kinase B (Akt), phospholipase C, gamma 1 (PLCγ1) and the transport of NF-κB into the nucleus. This inhibitory effect can also reduce inflammation of the skin, aorta, joints, liver, and neuronal tissues [16,44].

### 1.3. Anti-Tumor Effects of MEL

Given that MEL attacks lipid membranes and leads to substantial cytotoxicity, it has been widely studied in anti-tumor treatments (Table 2).

**Table 2:** Anti-tumor effects of MEL.

Tumor Type	Cell Lines	Treatment	Result or Mechanism	Reference
Lung cancer	A549 and NCI-H460 cell	MEL	IC_50_ values were 2 μg/mL, 3 μg/mL, respectively	[45]
	A549 cell	Antinucleolin aptamer–MEL conjugate	Viability for A549 cells after treatment was 51.2 ± 3.5%,	[46]
Hepatocellular carcinoma	SMMC-7721 cells	MEL	MEL inhibits G0/G1 cell cycle progression by down-regulating MeCP2 through Shh signaling.	[47]
	HepG2 cells	MEL	HDAC2-mediated PTEN upregulation, Akt inactivation, and inhibition of PI3K/Akt signaling pathways.	[48]
	SMMC-7721 and BEL-7402 cells	MEL	MEL sensitizes human hepatocellular carcinoma cells to tumor necrosis factor-related apoptosis-inducing ligand (TRAIL)-induced apoptosis by activating CaMKII-TAK1-JNK/p38 and inhibiting IκBαkinase-NFκB.	[49]
Breast cancer	MDA-MB-231 cells	MEL	MEL inhibits the EGF-induced MMP-9 expression via blocking the NF-κB and PI3K/Akt/mTOR pathway	[50]
	SUM159 and SKBR3	BV or MEL	MEL reduces the level of the PD-L1 immune-checkpoint protein and the immune-suppressive effects of the tumor microenvironment. IC_50_ values for MEL was 4.24 ng/μL for SUM159 and 3.59 ng/μL for SKBR3.	[51]
Prostate cancer	LNCaP, DU145, and PC-3 cells	BV or MEL	MEL induces cell apoptosis by activating the caspase pathway via NF-κB inactivation. IC_50_ for LNCaP cells: MEL 2.9 and BV 14.2 µg/mL, DU145 cells: MEL 1.5 and BV 6.3 µg/mL IC_50_ for PC-3 cells: MEL 1.8 and BV 6.1 µg/mL, respectively	[52]
Leukemia	CCRF-CEM and K-562 cells	MEL	MEL induces apoptosis via the intrinsic/mitochondrial pathway.	[53]

BV suppresses COX-2 mRNA expression and PGE 2 synthesis; hence, MEL may also exert anti-tumor effects [54]. MEL inhibits proliferation of the cancer cells via induction of apoptosis through multiple investigated mechanisms. One possible mechanism is that MEL causes changes in the permeability of cell membranes, which leads to the elevation of intracellular Ca^2+^, an important regulator in the apoptosis process, and activation of PLA2 [55], resulting in cell death. MEL presents a significant anti-tumor effect through the NF-κB pathway, which is involved in multiple physiological processes including tumor [56]. Its other mechanism of apoptosis includes decreasing methyl-CpG binding protein 2 [47] and PI3K/Akt/mTOR signaling pathway [48,50].

MEL also inhibits the invasion and metastasis of cancer cells. MEL prevents hepatocellular carcinoma cell metastasis via inhibition of ras-related C3 botulinum toxin substrate 1 (Rac1) [57], which participates in the c-Jun N-terminal kinase (JNK) and JNK-dependent cell motility processes and induces metastasis. In addition, MEL selectively inhibits expression of matrix metalloproteinase-9 (MMP-9), which plays an important role in the migration of cancer cells [50,58,59] via down-regulating activator protein-1 (AP-1) and NF-κB expression.

### 1.4. Obstacles to the Applications of MEL

Despite its multiple pharmacological potentials, the clinical applications of MEL are limited due to its strong surface activity and cytotoxicity. MEL results in 50% hemolysis of human red blood cells at 2 μM concentration and 100% at 7 μM [60]. Studies examining the antimicrobial activity of MEL in vivo [61] have found an increased mortality rate in mice directly injected with MEL. LD_50_ value of intraperitoneal MEL to mice is around 5 mg/kg, and drops to about 3 mg/kg via intravenous injection [62,63,64]. Preclinical and clinical research on BV therapy has indicated that the main adverse reactions of MEL include allergic reactions and pain at the administration site [65,66], which limits BV’s application in acupuncture therapies. Further applications of MEL are also restricted due to its degradability [67], low bioavailability [68], and non-specific lytic effects.

**Figure 2:**
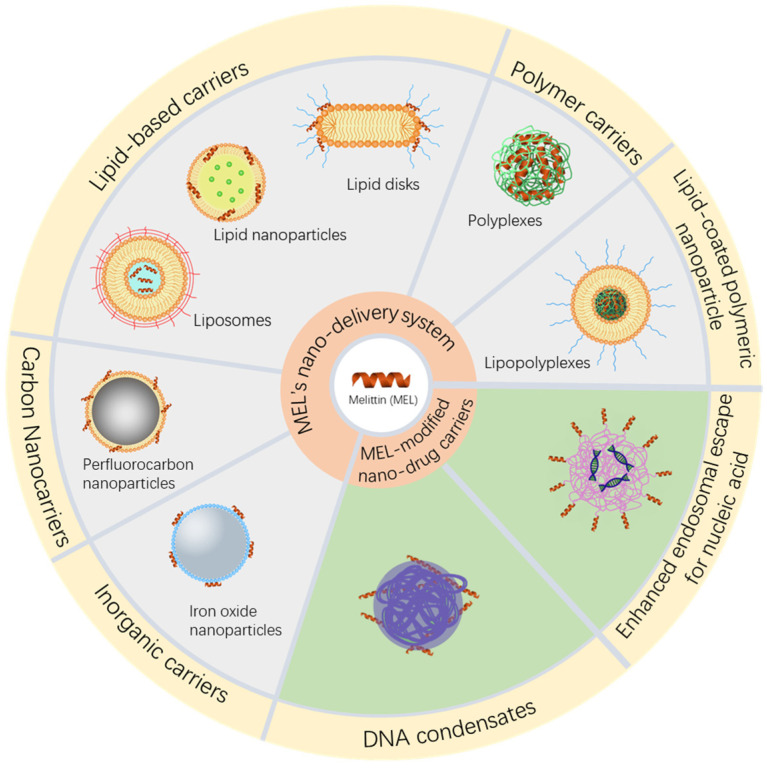
Strategies of MEL nano-delivery systems and MEL-modified nucleic acid nanocarriers for cancer therapy.

Multiple strategies have been applied to stably and safely load MEL to obtain effective anti-tumor as well as other therapeutic effects. The designated purposes of MEL delivery systems are approximately the same. Other than increasing the delivery efficiency of MEL, delivery systems conceal MEL to prevent it from interacting with cell membranes as well as to mask its positive charge so as not to bind to other proteins in vivo. Additionally, delivery systems enable targeting delivery to the lesion areas via the enhanced permeability and retention (EPR) effect or stimulus responsive designs. With surface activity and affinity for lipid membranes, MEL is also applied in drug delivery system to enhance cell uptake and endosomal escape (Figure 2).

## 2. Delivery Vehicles for Melittin

The following two main strategies are adopted to overcome the cytotoxicity and hemolysis effects of MEL: incorporating nanoparticles that can safely deliver a substantial amount of MEL through the intravenous route, and modifying MEL to reduce its toxicity.

### 2.1. Modified MEL and Conjugates

Changing the amino acid sequence of MEL or linking it with polypeptides or other molecules provides it with certain properties, such as in vivo stability or targeting. The hemolysis of MEL decreases after phosphorylation [69] or modification of amino acid sequences to obtain MEL analogs [70,71] has been proved. The derived MEL peptides have been designed and developed to exert more desirable properties, such as lower hemolysis effects [72,73,74], enhanced therapeutic properties [75,76] and even controllable activation providing targeting ability to tumor tissue [70,77]. Substituting alanine for leucine drastically reduces the hemolytic activity on human red blood cells, which is only about 1–2% of the hemolytic activity of MEL, while the antibacterial activity remains equivalent to MEL [73]. Meanwhile, modifications on the C-terminal region (R22A, K23A, R24Q) reduce the net charge of the protein and increase its pore-forming ability (20-fold more potent than MEL) [78].

In one study of MEL conjugate development, 2,3-dimethylmaleic anhydride (DMMA) was used to modify the active amino groups in MEL to obtain ultra pH sensitivity [79]. DMMA concealed the original positive charge of MEL and significantly reduced the hemolysis and clearance of reticulo-endothelial system. Other modification methods include immunoconjugates [46,80] and hybrid peptides [81]. MEL covalently links to the anti-nucleolar protein aptamer AS1411 and forms a conjugate that can achieve targeted delivery to multiple cancer cells. Compared with free MEL, this modified MEL has significantly reduced hemolytic activity and greater cytotoxicity in A549 cells [46]. The bacteria-killing efficacy of MEL is improved after being decorated on graphene (Gra) or graphene oxide (GO) nanosheets, which increase its ability to permeate the cell membrane and cause rapid bacterial leakage [82].

### 2.2. Nano Delivery Vehicles

Nano drug delivery systems have been widely applied in MEL delivery. Attempts such as inorganic carriers (including quantum dots, Fe_3_O_4_ nanoparticles, and perfluorocarbon nanoparticles), polymer carriers (including PLGA nanoparticles and β-cyclodextrin nanoparticles), and lipid carriers (including lipid disks, lipid nanoparticles, MEL–lipid conjugate nanoparticles and liposomes) greatly reduce the toxicity of MEL and provide the possibility of targeting to the intended sites (Table 3).

#### 2.2.1. Inorganic Carriers

Quantum dots are small semiconductor particles (size of a few nanometers) with unique optical and electronic properties as well as potential anti-tumor and photosensitive properties [106,107]. Dang et al. [83] modified CdSe/ZnS core/shell quantum dots by using the high-affinity interaction between phosphorylcholine and MEL. A fluorescence resonance energy transfer (FRET) system was formed between the Cy3b label on MEL and the quantum dots. This system was used to study the interaction between protein and membrane, and has potential applications in cancer therapy. The tumor-targeting and anti-tumor effects of quantum dots were demonstrated in lung cancer [108,109] and pancreatic cancer cells [110]. However, it is only in primary stage and cannot stably carry MEL for a long time to avoid MEL hemolysis.

Studies have proved the feasibility of loading MEL on inorganic metal nanoparticles such as iron oxide [111,112] and gold nanoparticles [113,114]. Hematyar et al. [84] developed a magnetic-responsive co-delivery system for effective cancer therapy. Doxorubicin (DOX) and MEL were loaded onto the surface of citric acid-functionalized Fe_3_O_4_ magnetic nanoparticles (CA-MNPs) through electrostatic interaction. CA-MNPs possess superparamagnetic nature and have potential to be directed and localized to tumor targets by external magnetic fields. The advantage of metal nanoparticles as MEL vectors is that several metal materials possess stimulus-responsive ability, which enhances targeting transportation of MEL, while some metal-based (gold or silver) nanoparticles have been proven to exert anti-cancer effects [115], and may obtain a better therapeutic effect in combination with MEL.

#### 2.2.2. Carbon Nanocarriers

Perfluorocarbon (PFC) nanoparticles are composed of a hydrophobic PFC core surrounded by a phospholipid monolayer where MEL can be stably inserted into the phospholipid monolayer without destroying the nanoparticle structure [70,85,116]. The toxicity of MEL to sperm and vaginal epithelial cells is reduced fivefold when delivered in PFC nanoparticles; to some extent, this characteristic guarantees the safety of MEL as an anti-HIV agent [116]. PFC nanoparticles extends the half-life of MEL in plasma from 24 min to more than 300 min and improves safety by promoting the clearance of circulating peptides through the reticuloendothelial system [86]. However, the PFC-containing MEL nanoformulation has a large particle size (250~300 nm), which may not be conducive to diffusion and cellular uptake on sites [117].

#### 2.2.3. Polymer Carriers

Poly (d, l-lactic acid-*co*-glycolic acid) (PLGA) is a common biodegradable polyester that can carry MEL in nanoparticles [87,88,89,118]. Yang et al. [88] paired MEL with the anionic agent sodium lauryl sulfate. The formed complex was highly soluble in organic solvents and formulated into MEL-PLGA nanoparticles, resulting in improved drug loading efficiency (~90%) and drug content (6–7%). The in vivo experimental study of Jeong et al. [87] on PLGA-coated BV preparations for pain inhibition showed that PLGA-coated MEL induced by acupuncture therapy significantly prolonged the time of pain suppression in rats and decreased the side effects by reducing the rate of release from nanoparticles. The MEL-loaded PLGA microspheres produced in high encapsulation can achieve a controlled release rate correlated with polymer degradation rate [119]. However, MEL-polymer nanoparticles formed based on charge interaction face obstacles in the stable loading of MEL under relatively complicated physiological conditions, and remain unsuitable for systemic circulation.

β-Cyclodextrin (β-CDP), an easily available polymer material, has a ring-shaped truncated cone topology with a hydrophobic cavity to non-covalently contain diverse guest molecules [120]. Xu et al. [90] established a library of self-assembled MEL nanoparticles based on β-CDPs and functional monomer adamantane derivatives (Ad-Ds). The cytotoxicity of 30 μg/mL MEL with 2 mmol/mL nanoparticle decreased by sixfold compared with that of free MEL.

#### 2.2.4. Lipid-Based Carriers

Lipodisk (or lipid disk) is a flat circular lipid bilayer structure where PEGylated lipid forms the highly curved edges of the lipodisk. Owing to the large curvature of the edge and hydrophobic interactions, MEL has high affinity with the edge of the lipodisk that allows it to bind to the disk preferentially; the interaction between MEL and PEG chains is negligible [91,121]. Gao et al. [92] established a cyclic RGD peptide (c (RGDyK))-modified lipid disk as a MEL carrier. In vivo experiments suggested that the lipodisk loaded with MEL significantly reduced the hemolysis effect and effectively inhibited tumor growth in mice. c (RGDyK) modification of the lipodisk increases its distribution in solid tumors and its anti-cancer efficiency. Ahlgren et al. [93] reported that EGF-targeted lipodisks had high MEL-loading efficiency and improved the specificity and cytotoxicity of MEL on tumor cells. Although the co-loading of MEL and paclitaxel in lipodisks did not induce hemolysis, the lipodisks loaded with MEL alone exhibited hemolytic toxicity at high concentrations [94], which may imply that the safety of lipid disks loaded with MEL needs to be further improved.

Modified MEL can be stably loaded into lipid nanoparticles (LNP). The hemolytic properties of MEL were concealed when linked to an amphipathic peptide, and then MEL interacted with phospholipids, self-assemble into lipid nanoparticles with a size of 15 nm [81]. With high MEL encapsulation rate (>80%) and neutral zeta potential, this LNP has a significant tumor inhibitory effect on melanoma cancer models, with an inhibitory rate of 82.8%. Other attempts to load MEL on nanoparticles include the use of a peptide–phospholipid scaffold to form an ultrasmall (10–20 nm) MEL-lipid nanoparticle (α-MEL-NP) [95,122] that targets lymph nodes and elicit an anti-tumor effect and immune response as a nanovaccine. α-MEL-NPs promote the release of whole-tumor antigens in situ. On the other hand, the size of α-MEL-NPs is optimal so that they can efficiently drain into lymphatic capillaries and lymph node, activating resident antigen-presenting cells [95]. As researches have proved a significant increase of affinity of MEL with positively curved lipid surfaces [123,124], both lipodisks and ultrasmall lipid nanoparticles increase the drug loading efficiency and loading stability, and has improved biocompatibility.

Liposomes are closed vesicles with a bilayer structure formed when phospholipid or phospholipid-like substances are dispersed in the aqueous phase [125,126]. MEL is amphiphilic, has a positive charge, and can be loaded into the aqueous phase of liposomes [97]. However, necessary measures must be taken to overcome the interaction between MEL and lipid layer to protect the liposome membrane from this cell-penetrating peptide. The nonionic block linear copolymer poloxamer 188, is applied in the preparation of MEL liposomes to prevent leakage [98,127], resulting in decreased hemolysis and MEL-induced vascular irritation. Mao et al. [98] attached poloxamer 188 to MEL in order to encapsulate MEL and form liposomes. In vitro experiments revealed that this material has a significant inhibitory effect on the survival of hepatocellular carcinoma (HCC) cells and suppresses the growth of subcutaneous and orthotopic liver cancer transplantation tumors in vivo. A recent attempt used a liposome modified with dioleoyl-phosphoethanolamine (DOPE)-coupled HA (HA-DOPE) to deliver MEL; the HA layer on the surface of liposome entraps MEL and prevents it from leakage [99]. However, the outer-surface modification of liposomes may exert a limited effect in preventing MEL from affecting the lipid bilayer structure of liposomes. Further improvement is needed for long-term circulation in vivo.

#### 2.2.5. Lipid-Coated Polymeric Nanoparticles

With the advantages of both the liposomes and polymeric nanoparticles, lipid-coated nanosized drug delivery systems have properties such as high drug loading capacity, high stability and biocompatibility, and prolonged circulation time in vivo [128], which make them promising for targeting delivery of MEL. Ye et al. [100] prepared a nanoparticle inner core with negative charges containing MEL and poly γ-glutamic acid (γ-PGA), an anionic polymer. The core is then coated by the cationic lipid to form liposomes, which effectively prevent the leakage of MEL. The outer shell is composed of PEG and PEG-targeting molecule (DSPE-PEG-RGD), providing stability in long-term circulation, capacity of selective binding with target tumor cells and cytolytic activity via apoptosis induction.

#### 2.2.6. Stimulus-Responsive Delivery Systems

Multiple stimulus-responsive nano delivery systems have been used to deliver MEL to attenuate the cytotoxicity of MEL during systemic circulation and to achieve its targeted delivery, including pH-responsive [101,102,103], magnetic-responsive [84], photosensitive [103,104] and redox-sensitive [105] delivery systems. The stimulus-responsive carrier maintains a stable state during circulation in the body and is stimulated and changes conformation or structure when the nanocarrier reaches the target site and releases MEL to a therapeutic concentration [84,101,104]. Similar to pH-sensitive MEL, the pH-responsive polymer is converted in a lower pH environment and releases active MEL. Lai et al. [101] developed a nanoparticle consisting of nanodiamonds and PEGylated polyglutamic acid, which exhibited enhanced cytotoxicity towards MCF-7 cells. A near-infrared (NIR) laser irradiation responsive nanosystem can be assembled using MEL, NIR-absorbing molecule cypate, and HA [103], of which size and morphology transform successively under changes in pH and NIR laser irradiation.

## 3. Nano Drug Delivery System with Melittin as a Functional Molecule

### 3.1. Melittin Enables Efficient Vesicular Escape

The entry of exogenously applied nuclear acid into the cytoplasm and its subsequent transport into the nucleus is a major cellular barrier for nonviral gene delivery vectors. A variety of strategies have been applied with the purposes of targeted delivery to the target sites and improved cellular uptake and endosome release, including polymer-or lipid-based nanoparticles [129] and liposomes [130] etc. As a cell-penetrating peptide, MEL can improve the cellular uptake of therapeutic compounds and the endosomal escape of nanoparticles [131,132]. With its strong surface activity, MEL can work as a penetrating peptide to promote escape from endosomes, thus increasing the bioavailability of nanoparticles [133]. Such features makes MEL popular as an oligonucleotide transfection agent [5], as the intracellular delivery of gene has always been a technical obstacle to overcome urgently [134].

The endosomal escape effect of conjugate of MEL with commonly used polymers for nucleic acid delivery has been widely verified. Transfection experiments on a variety of cell lines have shown that the transfection efficiency of MEL-PEI-luciferase DNA conjugates is up to 700-fold higher than that of controlled group (PEI-DNA conjugates) [133]. Ogris et al. [133] developed a conjugate that covalently attached MEL to poly (ethylenimine) (PEI) condensed DNA into small, discrete particles (<100 nm in diameter). Compared with PEI, the transfection activity of this conjugate was strongly increased within a broad range of cell lines and types. The connection between MEL and polymer has also been explored. PEI and MEL binding experiment [135] showed that the conjugate connected to the N-terminal of MEL (N-mel-PEI) has a low toxicity and a high transfection efficiency, whereas the PEI bound to the C-terminal of MEL (C-mel-PEI) shows a high cytotoxicity. A possible explanation is that the hydrophobic N-terminal of C-mel-PEI is easily inserted into the cell membrane and facilitates water flow into the bilayer, leading to membrane disturbance and instability. By contrast, N-mel-PEI tends to form a parallel conformation. This speculation is not universal; a stearyl attached to the C-terminus of MEL (stearyl-rMel) shows great efficiency, and the stearyl-rMel/p53 plasmid complex exhibits high p53 expression and anti-tumor activity [136].

### 3.2. Enhanced Drug Delivery of MEL as an Adjuvant

MEL has been widely studied and combined with various polymers to develop new, efficient, and safe non-viral gene drug delivery systems. In addition, studies reveal that MEL is able to act as attractant for certain receptors, such as PLA2 [38,137], for nano drug modification.

The problem with the use of MEL as an endosomal penetrating peptide is its non-specificity to the lipid membrane; the nanocarrier directly inserted with MEL will present a major safety risk. Therefore, MEL must be masked until it reaches the target sites. Oude Blenke et al. [138] explored the coupling of MEL to liposomes via functionalized PEG-lipids following aldehyde–hydrazide chemistry. At endosomal pH, the acid-labile hydrazone bond hydrolyzes and releases the peptide. Similarly, MEL can be applied in the conjugates of siRNA with pH-sensitive polymers by masking it with pH-labile dimethylmaleic anhydride (DMMAn) [139,140,141], or concealing it in micellar structure consisting of pH-sensitive poly (2-diisopropylaminoethyl methacrylate) (p (DIPAMA)) [142,143]. Another alternative strategy is to modify the peptide to reduce cytotoxicity. Changes in hemolytic activity can be achieved by MEL derivative peptides including pH-sensitive peptides [144,145], and light-sensitive peptides [146]. Chen et al. [147] transformed MEL into a sulfhydryl polymerized peptide and incubated with plasmid DNA to obtain peptide DNA condensates. The hemolytic potency of poly MEL was efficiently covered when combined to DNA. Modified MEL peptide p5RHH (sequence VLTTGLPALISWIKRKRQQ) transfects siRNA with an IC_50_ as low as 25 nM and possesses minimal cytotoxicity at the highest tested dose (10 μM) [71,148]. p5RHH also works as a linker inserted into nanoparticles and liposomes, and incorporates targeting ligands, imaging agents, and therapeutic drugs into particles without affecting their integrity [149].

The immune effects of MEL make it an effective adjuvant for vaccines. Owing to its ability to increase IFN-γ and IL-1β and decrease IL-10, MEL was selected as suitable adjuvant candidate for *Helicobacter pylori* intranasal vaccine development [150]. After deletion of the last four residues of the C-terminal region, with the aim of lowering the interactions with the cell membranes, the derived MEL peptide was linked to epitopes as an adjuvant. As the vaccine is administrated through the nasal mucosa, the side effect of MEL is largely eliminated. Another study showed an enhanced absorption effect of MEL as a mucosal adjuvant [151]. Compared with the control group and those receiving intranasal administration of free antigen, BALB/c mice administered with 4 μg of MEL and tetanus toxoid/diphtheria toxoid remarkably enhanced the antibody titers and prolonged the immune responses.

## 4. Conclusions and Prospect

MEL is a polypeptide with various pharmacological properties and has great potential in anti-inflammatory, anti-tumor, and anti-viral applications. Although pure MEL has toxicity and hemolytic properties, the use of protection or packaging greatly reduces its systemic toxicity. New strategies based on MEL can deliver drugs safely and effectively in the body. The modified peptide transduction domain and MEL-based technology are specifically designed to enhance endosomal escape and enable new nanoscale strategies to complete the arduous task of in vivo drug delivery. Upcoming designs such as stimulus responsiveness and masking are adopted in the transportation and functionalization of MEL.

However, challenges with respect to MEL-based nanodrug delivery systems still exist. Current methods cannot completely avoid the side effects of MEL, and the transfection efficiency of gene delivery systems still has room for improvement. Further understanding the mechanism of interaction of MEL with membrane and endosomal escape is crucial for the development of its delivery system.

Despite the limitations and challenges of MEL delivery systems, the development of various novel delivery strategies and clinical trials for various diseases is being undertaken to explore an ideal delivery system for MEL. The main directions of advancement are to design and synthesize modified MEL with weakened toxicity while maintaining its pharmacological effects, and to develop more stable and biocompatible delivery systems with improved targeting and controlled releasing ability. Other measures focused on MEL treatment include construction of tumor targeted gene vector containing MEL coding sequence that induced tumor cell-specific MEL expression.

## Figures and Tables

**Table 1:** In vitro anti-microbial effects of MEL.

Type of Microbial		Treatment or Method	Result	Reference
Virus	HIV-1	MEL	ID_50_ values was in the range 0.9–1.5 μM	[17]
	HSV-1 and HSV-2	MEL	CC_50_ ranges 1.35–2.05 μM	[18]
	SARS-CoV-2	Sitagliptin-MEL nano-conjugate	IC50 values 8.439 μM	[19]
Bacteria	*Pseudomonas aeruginosa*	MEL	MIC 10 µg/mL and MBC 20 µg/mL	[20]
	Methicillin-resistant *Staphylococcus aureus*	MEL	MIC 6.7 μg/mL and MBC 26 μg/mL.	[21]
	Multidrug-resistant *Acinetobacter baumannii*	MEL	MIC ranges 0.50–32 μg/mL	[22]
	*E. coli* and *Staphylococcus aureus*	MEL and ionic liquids combination	*E. coli*: MIC value was 0.52 μM MEL with 10 μM [Pyr C_12_]Br^−^ *S. aureus*: MIC value was 0.62 μM MEL with 20 μM [Pyr C_10_] Br^−^.	[23]
	Multidrug-resistant *Acinetobacter baumannii* and *Pseudomonas aeruginosa*	Combination of MEL and conventional antibiotics	MDR *A. baumannii* isolates: MIC for MEL and doripenem were reduced by 61.5 and 51.5 folds, respectively. MDR *P. aeruginosa* isolates: MIC was reduced by 63.5 and 58 folds for MEL–doripenem, respectively, and by 16 and 11 folds for MEL–ceftazidime, respectively.	[24]
Fungus	*Aspergillus flavus*, *Aspergillus fumigatus*, and *Aspergillus parasiticus*	MEL	MIC values was 1.25 μM, 1.25 μM, and 2.5 μM for *Aspergillus flavus*, *Aspergillus fumigatus*, and *Aspergillus parasiticus* strains respectively.	[25]
	*Candida albicans*	MEL	MIC values for different strains of *Candida albicans* ranges from 8 μM to 32 μM.	[26]

**Table 3:** Summary of MEL-loading nano-delivery systems and applications.

Type	Loading Strategy	Size	Applications	Reference
Quantum dots	MEL was modified to CdSe/ZnS core quantum dots	5–10 nm	Quantum dots were used to study the interaction between protein and membrane, and had potential to deliver MEL in vivo.	[83]
Iron oxide nanoparticles	MEL and doxorubicin (DOX) were co-loaded to citric acid-functionalized Fe_3_O_4_ magnetic nanoparticles (CA-MNPs)	20 nm	The release of both MEL and DOX was strongly enhanced at pH 4.5 and the nanoparticles were potentially applied in magnetically targeted cancer therapy.	[84]
Perfluorocarbon (PFC) nanoparticles	MEL was added to the PFC nanoparticles	~290 nm	PFC nanoparticles retained their structural integrity after the addition and contribute to the stability and slow dissociation of MEL from the stabilizing monolayer.	[85]
	MEL was mixed and incubated with PFC nanoparticles	227 nm	The growth of the tumors was inhibited by 24.68% in MDA-MB-435 human breast cancer.	[86]
	MEL derivative peptide was incubated with PFC nanoparticles composed of egg phosphatidylcholineand dipalmitoylphosphatidylglycerol	~280 nm	This MEL derivative is activated by matrix metalloproteinase-9 (MMP-9), a protease overexpressed in many tumor cells. In addition, treatment of PFC nanoparticles resulted in ~54% reduction in melanoma tumor size in vivo.	[70]
Poly (d,l-lactic acid-coglycolic acid) (PLGA) nanoparticles	BV-loaded PLGA/PVA nanoparticles	180 nm	PLGA nanoparticles reduced side effects by slowing down BV release, and prolonged suppression of nociceptive behavior in rats with formalin-induced pain.	[87]
	MEL was modified with sodium dodecyl sulfate and then formulated into PLGA nanoparticles	~130 nm	MEL was loaded with a high encapsulation efficiency in the nanoparticles and the concentration of half the cell growth (GI_50_) in breast cancer MCF-7 cells was 4.42 μg/mL in vitro.	[88]
	Tetrameric MEL binds avidly to PLGA-NPs	110 nm	Biodegradable tetrameric MEL is encapsulated in nanoparticles at efficiency of 97% and retains lytic activity.	[89]
β-cyclodextrin(β-CDP) nanoparticles	5 different functional monomer adamantane derivatives (Ad-Ds) incubated with β-CDPs respectively, and then mixed with MEL	30–200 nm	The percentage of hemolytic toxicity neutralization reached 100% at the concentration of 100 μM. The cytotoxicity of 30 μg/mL MEL with 2 mmol/mL nanoparticle decreased by sixfold compared with that of free MEL in CCRF-CEM cells.	[90]
Lipodisks	MEL incubated with PEG-stabilized lipid disks which composed of POPC/cholesterol/ceramide-PEG_5000_	20–100 nm	PEGylated lipodisks allowed stable loading of MEL, and retained anti-bacterial activity of MEL in *E. coli*, but extended the actions by slowing down releasing rate.	[91]
	Lipid disks was modified by c(RGDyK)-PEG_3400_-DSPE	50 nm	The disks induced no hemoglobin release at maximum tested concentration (100 μg/mL) and presented significate targeting and in vivo anti-tumor effect towards U87 glioma cells.	[92]
	MEL loaded lipodisks contained EGF-conjugated PEG-lipids.	~20 nm	The EGF-targeted lipodisks binded specifically to A-431 tumor cells, and resulted in a improved cell-killing effect, as cell viability decreased 20% compared to free MEL.	[93]
	MEL and paclitaxel were co-loaded within 9G-A7R modified lipodisks.	~50 nm	Co-loading prevented leakage of MEL from the disks and improved cytotoxicity on U87 cells in vitro and anti-tumor effect in intracranial glioma models. The synergistic effect of MEL and paclitaxel was proved as combination index values was 0.45.	[94]
Lipid nanoparticles	MEL was linked to an amphipathic peptide then loaded in ultrasmall lipid nanoparticles	14 nm	The ultrasmall lipid nanoparticles significantly reduced the hemolysis of MEL and showed obvious anti-tumor effect in malignant melanoma B16F10 cells, with IC_50_ values being 11.26 μM.	[81]
MEL-lipid conjugate nanoparticles	MEL-phospholipid scaffold	10–20 nm	The nanoparticles induced tumor cell apoptosis, releasing whole-tumor antigens in situ, and targeting to lymph nodes.	[95]
Liposomes	MEL was loaded in PEGylated anti-HER2 immunoliposomes modified by the complete antibody (trastuzumab)	139 nm	The immunoliposomes decreased cancer cells viability in a dose–response manner and in correlation to the level of HER2 expression in human breast cancer cells.	[96]
	MEL loaded liposomes was modified by antibodies against the fish viral hemorrhagic septicemia rhabdovirus (VHSV) glycoprotein G (gpG)	~140 nm	The in vitro antiviral studies showed that the liposomes inhibited the infectivity by 95.2% through inactivating VHSV.	[97]
	MEL was modified with 2% poloxamer 188 then loaded in nano-liposomes.	NA	Multiple hepatic carcinoma cell lines (Bel-7402, BMMC-7721, HepG2, LM-3, and Hepa 1–6 cells) were sensitive to the liposomes, and the IC_50_ value was close to free MEL, indicating efficient anti-tumor effect.	[98]
	Hyaluronic acid (HA) modified MEL-loading liposomes	133 nm	HA enhanced the sustained-release effect of MEL from the liposomes and provide targeting ability via specific binding with CD44, which is highly expressed on the surface of melanoma B16F10 cells.	[99]
Lipid-coated polymeric Nanoparticles	MEL and poly γ-glutamic acid (γ-PGA) formed nanoparticles which then coated by cationic liposomes modified by PEG and DSPE-PEG-RGD	~100 nm	The hemolytic activity and nonspecific cytotoxicity of MEL were remarkably reduced by the lipid-coated polymeric nanoparticles and the RGD-modified RGD modified nanoparticles effectively induced apoptosis in A549 cells.	[100]
Stimulus-responsive delivery systems	MEL was grafted to nanodiamonds coated with PEGylated PGA.	220 nm	The nanoparticles were pH sensitive and steady able to released MEL in an acidic environment. Toxicity to breast cancer MFC-7 cells was enhanced than free MEL in a concentration-dependent manner.	[101]
	D-MEL was conjugated with PEG which is polymerized with DIPAMA and PDSEMA, to form micelles.	33 nm	The pH sensitive micellar formulations unsheathes MEL only at endosomal pH, remarkably reducing hemolytic effects of MEL, and IC_50_ for the micelles in 3T3, A549, CT26 cancer cells were 8.5 μM, 6.9 μM, 11.6 μM, respectively.	[102]
	MEL was loaded in negatively charged nanospheres consisting of NIR-absorbing molecule cypate and HA.	∼50 nm	The nanospheres responsive to both pH and near-infrared (NIR) laser irradiation changes into net-like nanofibers and small nanospheres (~25 nm) when stimulated and induce cancer cell death, inhibit the metastatic dissemination of tumor cells, and facilitated deep tumor penetration o	[103]
	Serum albumin (SA)-coated boehmite scaffold was loaded with photosensitizer chlorin e6 (Ce6) and MEL.	184 nm	The nanocarrier exerted high encapsulation efficiency of MEL and low hemocompatibility. In vivo phototreatment of the scaffold eliminated 4T1 cells remarkably in subcutaneous breast tumor models.	[104]
	MEL loaded in redox-sensitive nanocomplexes	357 nm	The nanocomplexes decreased hemolysis of MEL and released MEL responding to high redox potential environment, and showed an enhanced cytotoxicity on both HCT 116 colon cancer cells and MCF-7 breast cancer cells.	[105]
